# Plasmon Enhanced Second Harmonic Generation from ZnO Nanofilms on Vertical Au Nanorod Arrays

**DOI:** 10.3390/nano11102597

**Published:** 2021-10-02

**Authors:** Qiang Ma, Chengda Pan, Yingxian Xue, Zhiyun Fang, Shiyu Zhang, Botao Wu, E Wu

**Affiliations:** 1 State Key Laboratory of Precision Spectroscopy, East China Normal University, Shanghai 200241, China; sniper0617@foxmail.com (Q.M.); 51162099017@stu.ecnu.edu.cn (C.P.); 52190920031@stu.ecnu.edu.cn (Y.X.); 51190920047@stu.ecnu.edu.cn (Z.F.); 51200920038@stu.ecnu.edu.cn (S.Z.); ewu@phy.ecnu.edu.cn (E.W.); 2 Collaborative Innovation Center of Extreme Optics, Shanxi University, Taiyuan 030006, China

**Keywords:** surface plasmon, vertical gold nanorod arrays, second harmonic generation, ZnO, finite-difference time-domain

## Abstract

Vertically aligned gold nanorod arrays have attracted much attention for their fascinating optical properties. Different from longitudinal surface plasmon wavelength (LSPW) and edge-to-edge spacing of gold nanorods, the role of gold nanorod diameter in plasmonic enhancement ability of vertical gold nanorod arrays has rarely been explored. In this work, we selected gold nanorods with similar LSPW but two different diameters (22 and 41 nm), the optical properties of which are dominated by absorption and scattering cross sections, respectively. The vertically aligned arrays of these gold nanorods formed by evaporation self-assembly are coupled with nonlinear ZnO nanocrystal films spin-coated on their surfaces. It was found that the gold nanorod array with a larger diameter can enhance the second harmonic generation (SHG) of ZnO nanofilm by a factor of 27.0, while it is about 7.3 for the smaller gold nanorod array. Theoretical simulations indicate that such stronger enhancement of the larger vertical gold nanorod array compared with the smaller one is due to its stronger scattering ability and greater extent of near-field enhancement at SHG fundamental wavelength. Our work shows that the diameter of gold nanorods is also an important factor to be considered in realizing strong plasmon enhancement of vertically aligned gold nanorod arrays.

## 1. Introduction

Local surface plasmon resonance (LSPR) is the collective oscillation of the conduction electrons on the surface of metal nanoparticles when interacting with light. It can localize and conduct light at sub-wavelength scale and produce near-field enhancement of up to several orders of magnitude in the nano-scale range near the surface of metal nanoparticles. This fascinating property has been widely used in various fields, such as plasmon enhanced chemical reactions and spectroscopy, chemical and biological sensing and photocatalysis [1,2,3,4,5,6,7,8]. The LSPR near-field enhancement of metal nanoparticles depends not only on the type, size and surrounding environment of metal, but also its shape is a key factor. Compared with isotropic metal nanoparticles, anisotropic metal nanoparticles have attracted much attention because of their smaller local mode volume and stronger local electromagnetic field enhancement.

Anisotropic Au nanorods exhibit transverse and longitudinal surface plasmon resonance that corresponds to electron oscillations perpendicular and parallel to the rod long axis direction, respectively. One of the most intriguing properties of Au nanorods is that their LSPR depends strongly on the nanorod aspect ratio and can be systematically tuned from the visible to infrared region [9,10,11]. Au nanorods have a large near-field enhancement due to the strong localization of the surface electrons at the rod tips, which have been used to realize plasmon enhanced single molecule fluorescence [3,5,12], and to enhance the luminescence of single quantum dot and single rare earth ion doped upconversion nanocrystals [6,13,14,15]. However, because the active plasmon enhancement only exists in the range of tens of nanometers on the surface of metal nanoparticles, how to locate molecules and fluorescent nanoparticles at Au nanorod tips is challenging. At present, a single molecule can be linked to Au nanorod tips through selective molecular modification [3], or a single nanoparticle can be located at Au nanorod tips through template assisted localization or atomic force microscope probe nanomanipulation [6,14]. However, these methods are either cumbersome or inefficient. Therefore, it is necessary to develop a convenient and rapid method to couple Au nanorod tips with molecules or fluorescent nanoparticles.

Recently, evaporation induced self-assembly of Au nanorods based on the coffee ring effect has attracted extensive attention as a convenient way to obtain large-scale ordered arrays of Au nanorod tips [16,17,18,19,20,21,22], which have been used to enhance Raman scattering and luminescence [23,24,25,26]. On the other hand, the near-field enhancement effect of Au nanorods is also related to their scattering ability, which depends on the diameter of Au nanorods [10]. For example, for Au nanorods with similar longitudinal surface plasmon wavelength, the absorption cross section of Au nanorods with smaller diameter such as 10 nm is larger than the scattering cross section, showing a strong photothermal effect, while for Au nanorods with larger diameter such as 30 nm, the scattering cross section is larger than the absorption cross section, showing a strong near-field enhancement effect, and these types of Au nanorods are used to enhance spectroscopy and biological imaging. Gold nanorods with various diameters (8.9~48 nm) have been successfully self-assembled into vertical Au nanorod arrays [16,17,18,19,20,21]. However, the influence of the scattering/absorption ratio of Au nanorods, that is, the diameter on the enhancement ability of vertical gold nanorod arrays has rarely been explored.

Nonlinear nanocrystals with sub-wavelength geometries, such as ZnO nanowires and nanofilms, BaTiO_3_, LiNbO_3_, ITO and KTP nanoparticles, can achieve higher frequency conversion efficiency without phase matching conditions compared with their corresponding bulks. Unfortunately, the conversion efficiency of these nonlinear nanocrystals is still low for their practical applications. In order to improve the conversion efficiency of nonlinear nanocrystals, enhancing the conversion efficiency of nonlinear nanocrystals by coupling with plasmonic metal nanostructures has been explored [27,28,29,30,31,32,33]. In this work, we selected Au nanorods with similar longitudinal surface plasmon wavelengths but two different diameters (22 and 41 nm), and their extinction properties are dominated by absorption and scattering cross sections, respectively. The evaporation self-assembly of the two Au nanorods was realized by adjusting the concentration of CTAB molecules in the solution, and large-area vertical Au nanorod multilayer arrays were obtained. ZnO nanocrystal films with a thickness of about 126 nm were coated on the two vertical Au nanorod arrays. The plasmonic enhancement of vertical Au nanorod arrays on the second harmonic generation (SHG) efficiency of ZnO nanofilms is explored and discussed. The results show that vertical Au nanorod array with a larger diameter (41 nm) can enhance the SHG of ZnO nanofilm by 27.0 times due to its strong scattering ability, compared to about 7.3 times for the smaller vertical Au nanorod array (22 nm). The theoretical simulation based on the finite-difference time-domain (FDTD) method shows that compared with the smaller Au nanorod array, the larger Au nanorod array has higher electric field enhancement at the SHG fundamental frequency and thus a stronger enhancement effect, which is consistent with the experimental results.

## 2. Materials and Methods

Au nanorods with two different diameters (22 and 41 nm) were purchased from NanoSeedz Corp. (Hong Kong, China), and their nominal average diameter and length as specified by the supplier were 20 × 51 and 40 × 96 nm, respectively. Two kinds of Au nanorods were dispersed in water with the same CTAB concentration of about 3.6 mM, and the concentrations of Au nanorods with the diameter of 22 and 41 nm were 7.6 and 29.7 nM, respectively. To assemble vertically aligned Au nanorod arrays, a small aliquot (20 µL) of Au nanorod solution with appropriate CTAB and Au nanorod concentrations was deposited on silicon wafer cleaned sequentially by DECON 90, milli-Q water, absolute ethyl alcohol and milli-Q water and finally dried by nitrogen, then left to evaporate in constant temperature and humidity for 24–48 h. The evaporation temperature and humidity were controlled at 20 °C and 90%, respectively. The vertical Au nanorod arrays were formed. Before being spin-coated with ZnO nanocrystals, vertical Au nanorod arrays were cleaned by plasma to remove organics on their surface. ZnO nanopowder purchased from Merck was dispersed in absolute ethyl alcohol with a concentration of 10 mg/mL, and then the ZnO solution was spin-coated onto the surface of vertical Au nanorod arrays at 3000 rpm for 60 s to form a ZnO nanocrystal film. Scanning electron microscopy (SEM) images of Au nanorods and vertical Au nanorod arrays were acquired using a field emission scanning electron microscope (Sigma 300, Carl Zeiss AG, Oberkochen, Germany) operating at 15 kV. Transmission electron microscopy (TEM) images of ZnO nanopowder were taken on a JEM-2100F transmission electron microscope (JEOL Ltd., Tokyo, Japan) operating at 120 kV, and X-ray diffraction analysis was performed on a diffractometer (SmartlabSE, Rigaku Co., Tokyo, Japan) using Cu/Kα1 (λ = 1.54056 Å) as the radiation to identify the ZnO crystal phase. Absorption spectra of Au nanorod solution were measured using a home-built setup with a halogen lamp (100 W) as an illumination source and a fiber optical spectrometer (PG2000-Pro-Ex) to collect spectrum data. Dark-field imaging of individual Au nanorods was carried out with an Olympus IX71 inverted optical microscope integrated with a camera (DFK 31AF03). A 100 W halogen lamp was used to illuminate Au nanorods through a dark-field condenser (NA 1.2), and the scattered light was collected with a 60× objective (NA 0.7) and reflected to the camera for imaging.

**Figure 1:**
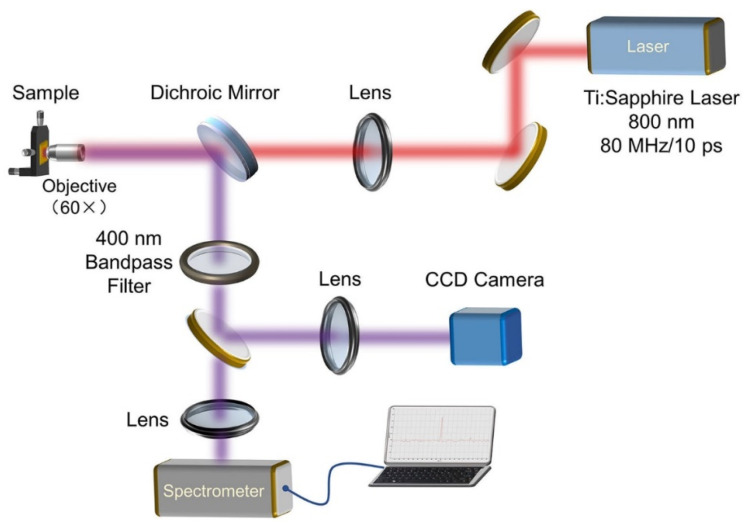
Experimental setup for the SHG measurement of ZnO nanofilms.

The SHG characteristics of ZnO nanofilms on vertical Au nanorod arrays were conducted using a home-built optical measurement setup based on the reflection configuration, as shown in Figure 1. A mode-locked Ti/sapphire laser (Tsunami, Spectra-Physics, Newport Co., Irvine, CA, USA; pulse duration 10 ps, repetition rate 80 MHz, @800 nm) was used to pump ZnO nanofilms along the perpendicular direction through a 60× objective (NA = 0.9). The back-reflected SHG signal was collected with the same method and transmitted to a CCD camera or a spectrometer (Andor SR500i, Oxford Instruments, Oxford, UK) for spectrum detection. In the front of the CCD camera and spectrometer, a 400 nm bandpass filter was used to remove the noise signal and incident laser. The laser power was measured using a laser power meter with a precision of 0.1 mW.

Absorption, scattering and extinction spectra of Au nanorods and near-field distributions of vertical Au nanorod arrays were simulated using commercial FDTD Solutions software (Version 2021 R1.3, Lumerical Solutions, Inc., Vancouver, BC, Canada). For the simulation of Au nanorod spectra, a total field/scattered field (TFSF) source ranging from 350 to 850 nm was illustrated. The ambient refractive index was set to 1.33. Perfected matched layers in *x*, *y* and *z* directions and a 3D nonuniform meshing with a 0.5 nm grid size in the total field domain were applied. The absorption and scattering cross sections were estimated by using a set of power monitors to calculate the net power flowing into the total and scattered field simulation domains, and their sum gives the extinction cross section. We constructed a three-layers vertical Au nanorod array with a 2 nm interlayer and edge-to-edge spacing of Au nanorods placed on a Si substrate to simulate its near-field distributions, which is illustrated by a plane wave source ranging from 350 to 900 nm. Periodic boundary conditions in both *x* and *y* directions and perfected matched layers in *z* direction were applied around the array unit cell, and the meshing grid size was also 0.5 nm. The electric field distributions at the fundamental and harmonic frequencies can be acquired in the power monitors. The experimentally measured Au and Si dielectric constants were chosen [34], and that of ZnO was set as 2.05. All simulations were tested for convergence.

## 3. Results

**Figure 2:**
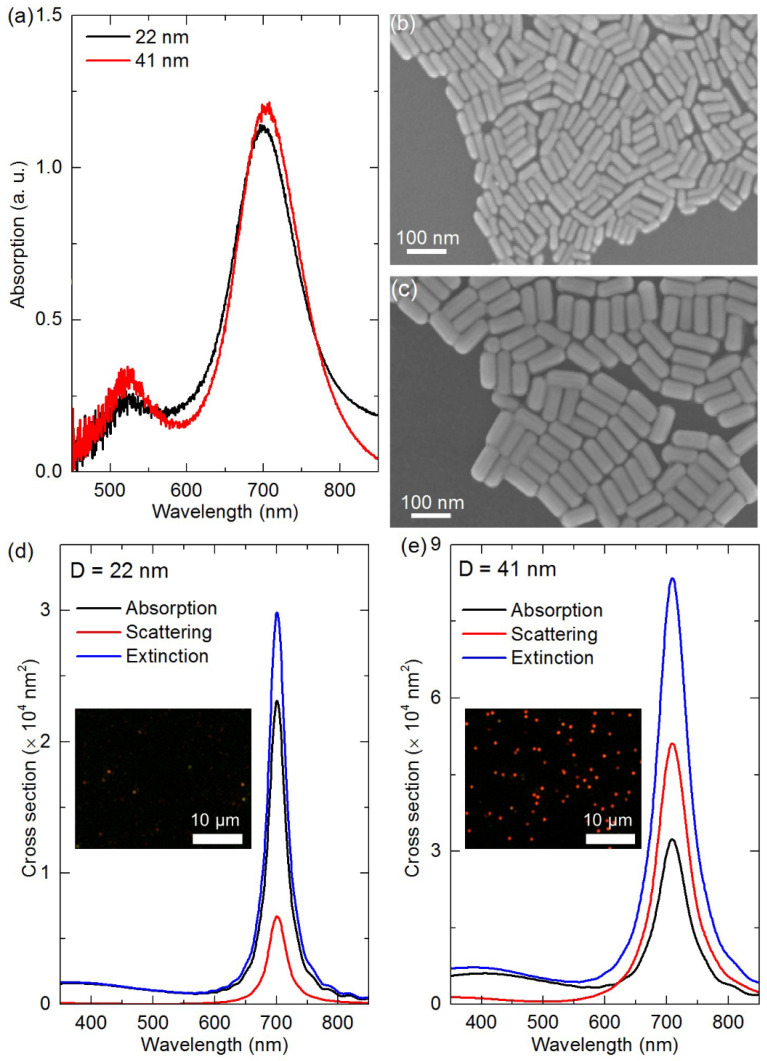
(**a**) Absorption spectra of Au nanorods with the diameters of 22 and 41 nm in water; (**b**) SEM image of Au nanorods with a 22 nm diameter; (**c**) SEM image of Au nanorods with a 41 nm diameter; (**d**) simulated absorption, scattering and extinction spectra for Au nanorods with a 22 nm diameter; (**e**) simulated absorption, scattering and extinction spectra for Au nanorods with a 41 nm diameter. The insets in (**d**,**e**) are dark-field scattering images of individual Au nanorods deposited on glass substrates.

Firstly, the optical and morphological properties of Au nanorods with different diameters for evaporation self-assembly were characterized. Figure 2a–c shows the absorption spectra and SEM images of two Au nanorods in aqueous solution. It can be seen that Au nanorods with different diameters have similar longitudinal surface plasmon resonance wavelengths at 702 nm. The SEM images in Figure 2b,c reveal that the diameter and length dimensions of the two Au nanorods are 22 ± 2 × 58 ± 5 nm and 41 ± 3 × 94 ± 7 nm, respectively. Based on the FDTD method, the absorption, scattering and extinction spectra of the two Au nanorods in water were simulated by using the above average sizes, as shown in Figure 2d,e. The simulated longitudinal surface plasmon resonance wavelengths of the two Au nanorods are all about 701 nm, which is consistent with the measured ones in Figure 2a. Because the two kinds of Au nanorods have similar aspect ratios (2.6 vs. 2.3), their measured and simulated longitudinal surface plasmon resonance peak wavelengths are similar. It should be pointed out that the average sizes of the two Au nanorods were used in the simulations, while the sizes of Au nanorods in solution were polydisperse, and thus the measured spectra of the two Au nanorods were all wider than the simulated ones. Moreover, the absorption cross section of small-diameter Au nanorods (22 nm) was larger than the scattering cross section, while for large-diameter Au nanorods (41 nm) the scattering cross section dominated, which can be confirmed by dark-field scattering imaging of individual Au nanorods deposited on glass substrates. The representative dark-field scattering images of Au nanorods with the diameters of 22 and 41 nm in the same measurement conditions are shown in the insets of Figure 2d,e, respectively. There are many red scattering bright spots from individual Au nanorods on the dark-field image of large-diameter Au nanorods, while there are only a few weak red spots for small-diameter Au nanorods, many of which are almost invisible. This clearly shows the strong scattering ability of large-diameter Au nanorods, consistent with the simulation results.

**Figure 3:**
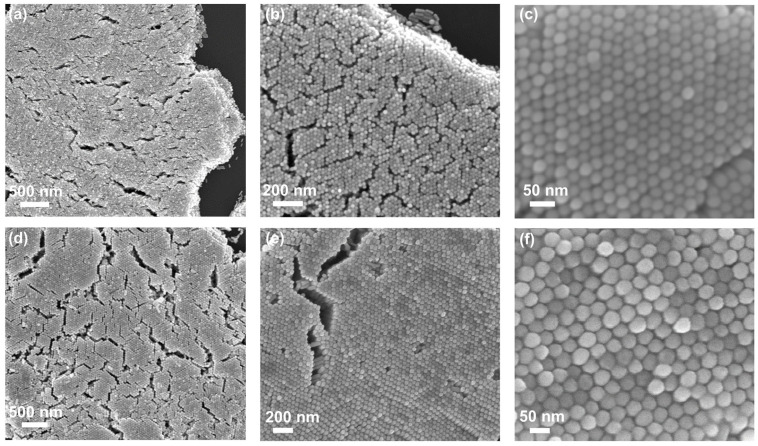
(**a**–**c**) SEM images of Au nanorods with a diameter of 22 nm at low, medium and high magnification; (**d**–**f**) SEM images of Au nanorods with a diameter of 41 nm at low, medium and high magnification.

Then, evaporation self-assembly based on the coffee ring effect was carried out by using these two kinds of Au nanorods [18,19,21,23,24]. The self-assembly of Au nanorods due to solvent evaporation occurs at ambient pressure and temperature, attributed to a balance between the attractive van der Waals interaction, capillary action and the repulsive forces between the CTAB molecules [22]. Through the systematic adjustment of the concentrations of Au nanorods and CTAB in the solution, it was found that for Au nanorods with a diameter of 41 nm, when the concentrations of CTAB and Au nanorods are about 1.8 mM and 15.0 nM, a large area of vertical Au nanorod array can be obtained, and the area of standing Au nanorods is more than 80%. For Au nanorods with a diameter of 22 nm, when the Au nanorod concentration is 7.6 nM and the CTAB concentration is about 2.5 mM, a large-area vertical Au nanorod array can also be obtained. Organic residues were randomly found on the surface of vertical Au nanorod arrays (Figure S1), so they were cleaned by plasma. Figure 3a–c and d–f show the SEM images of Au nanorods with the diameters of 22 and 41 nm after plasma cleaning at low, medium and high magnification, respectively. It can be noted that the plasma cleaning makes the array crack. It can be found from the array and crack edges that the multilayer vertical Au nanorod arrays are obtained by self-assembly. Similar to the previous work, the nanorods are organized into closely packed ordered hexagonal structures. From the high magnification SEM image (Figure 3c,e), the spacing between adjacent Au nanorods is about 2~3 nm, which is enough to produce strong enhanced electromagnetic near-field “hot spots” [24].

**Figure 4:**
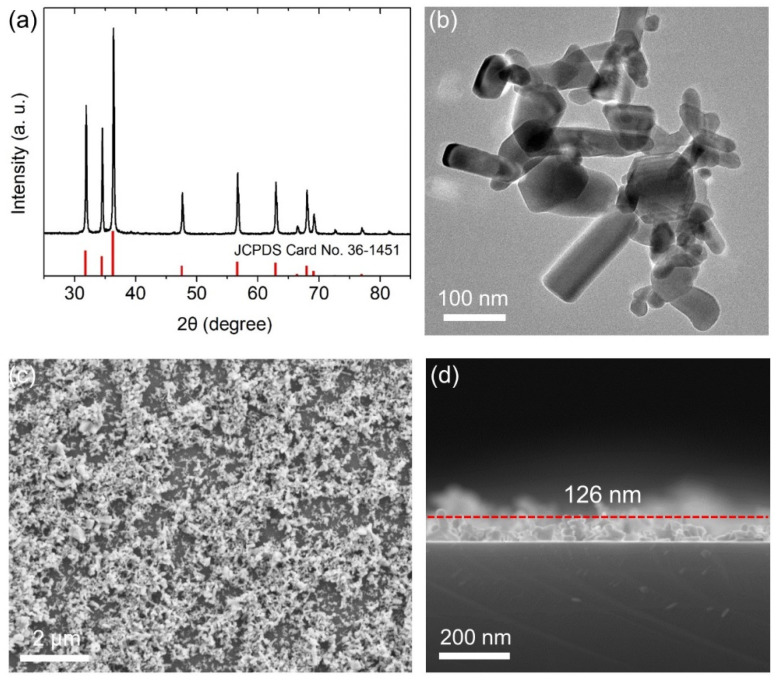
(**a**) XRD pattern of ZnO nanocrystals; (**b**) TEM image of ZnO nanocrystals; (**c**) SEM surface image of ZnO nanofilm; (**d**) cross section SEM image of ZnO nanofilm.

The characterization of nonlinear ZnO nanocrystals and its nanofilms is shown in Figure 4. As can be seen by the XRD pattern of ZnO nanopowder in Figure 4a, all the peaks can be well indexed by hexagonal wurtzite phase ZnO crystals (JCPDS file No. 36-1451), providing evidence of the pure crystalline nature of ZnO nanopowder. A representative TEM image of ZnO nanocrystals is shown in Figure 4b. Nanorods with varied sizes dominate as well as a small quantity of nanoparticles. From the XRD results, we estimate the average size (*d*) of ZnO nanocrystals by Scheler’s equation:

*d* = *kλ*/(*β*_0_cos(*θ*)), (1)

where *β*_0_ is the full width at half maximum of the diffraction, *θ* is the angle of diffraction, *λ* is the wavelength of X-ray and *k* is a constant. The diffraction peak around 2*θ* = 36.4° was selected for the calculation. The calculated average ZnO size is about 44.6 nm. ZnO nanopowder was dispersed into absolute ethanol, and ZnO nanofilm was formed on the vertical Au nanorod array by spin coating, as shown in Figure 4c. The average thickness of ZnO nanofilm can be estimated to be about 126 nm by the SEM image of the film cross section (Figure 4d).

**Figure 5:**
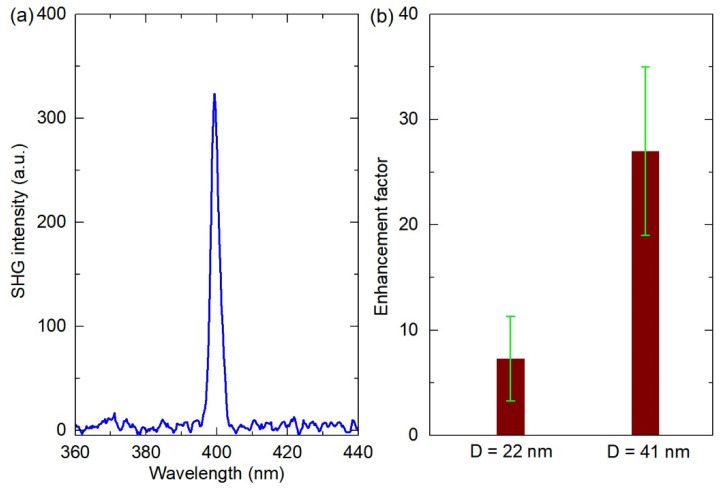
(**a**) Representative SHG spectrum of ZnO nanofilm; (**b**) SHG enhancement factor of ZnO nanofilms on the vertical Au nanorod arrays with the diameters of 22 and 41 nm.

Using the experimental setup shown in Figure 1, we studied the SHG optical nonlinearity before and after the coupling of ZnO nanofilms with vertical Au nanorod arrays. Figure 5a shows a representative SHG spectrum of ZnO nanofilms. To quantitatively evaluate the SHG enhancement efficiency of Au nanorod arrays with different diameters on ZnO nanocrystal films, the SHG enhancement factor *η*_EF_ was introduced by comparing the SHG intensity of ZnO nanocrystal films on vertical Au nanorod arrays (*I*_2*ω*(hybrid)_) and nearby bare ZnO nanocrystal films (*I*_2*ω*(bare)_):

*η*_EF_ = *I*_2*ω*(hybrid)_/*I*_2*ω*(bare)_. (2)

**Figure 6:**
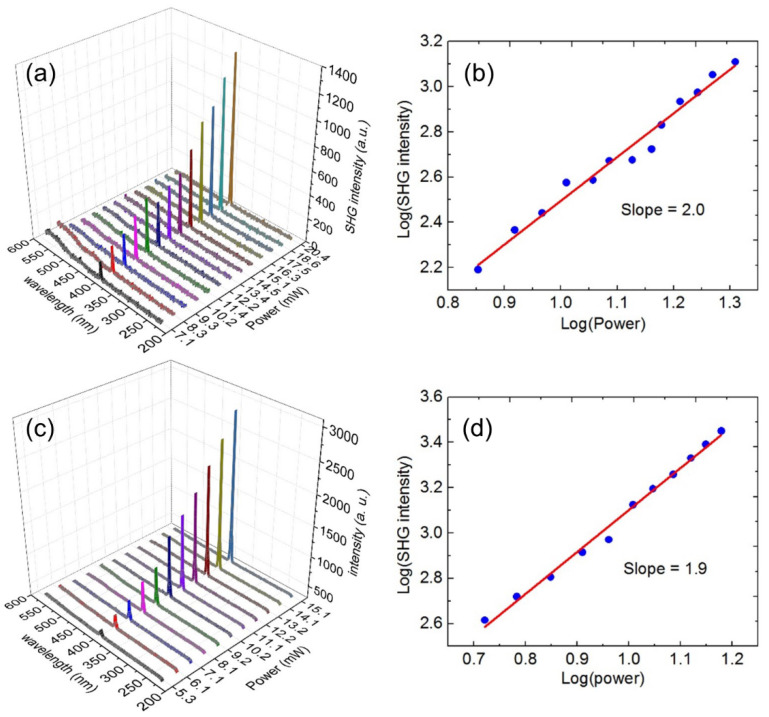
(**a**) SHG spectra at different excitation powers and (**b**) SHG intensity vs. excitation power in a logarithmic scale for the vertical Au nanorod array with a diameters of 22 nm; (**c**) SHG spectra at different excitation powers and (**d**) SHG intensity vs. excitation power in a logarithmic scale for the vertical Au nanorod array with a diameters of 41 nm.

At a given laser excitation power (10.2 mW), we measured 20 points for each vertical Au nanorod array sample with different diameters, and the resulting SHG enhancement factor is shown in Figure 5b. The vertical Au nanorod array with a larger diameter (41 nm) can enhance the SHG of ZnO nanofilm with an average SHG enhancement factor of about 27.0, while that of the vertical Au nanorod array with a smaller diameter (22 nm) is only about 7.3. That is, the SHG enhancement efficiency on ZnO nanofilm of the larger-diameter vertical Au nanorod array is about 3.7 times that of the smaller-diameter vertical Au nanorod array. Next, the dependence of SHG intensity of ZnO nanofilms on laser excitation power was studied, as shown in Figure 6. Figure 6a,c show the SHG spectra for the vertical Au nanorod arrays with the diameters of 22 and 41 nm at different excitation powers, respectively, and Figure 6b,d show the plots of the corresponding SHG intensity vs. excitation power in a logarithmic scale. The slopes of the fitting lines for the Au nanorod arrays with the diameters of 22 and 41 nm are 2.0 and 1.9, respectively. The quadratic response of the SHG intensity as a function of the incident laser power verifies a second harmonic nonlinear process in our experiments [27,29,30,31,33].

**Figure 7:**
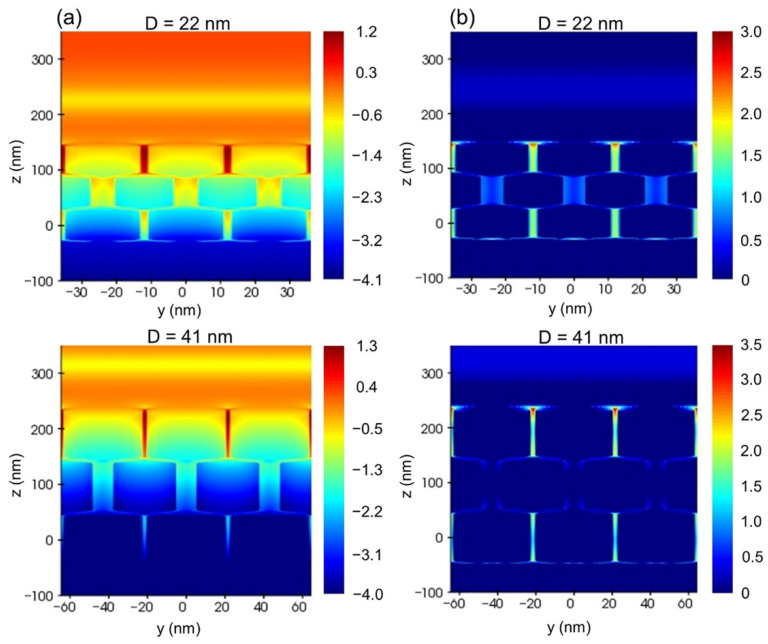
Simulated near-field |*E*/*E*_0_|^2^ distributions of vertical Au nanorod arrays with the diameters of 22 and 41 nm at the logarithmic scale: (**a**) harmonic wavelength (400 nm); (**b**) fundamental wavelength (800 nm).

To further understand the internal physical mechanism of SHG enhancement by Au nanorod arrays with similar longitudinal surface plasmon resonance wavelengths but different diameters, we conducted FDTD simulation at fundamental and harmonic wavelengths (400 and 800 nm) and calculated near-field distribution images, as shown in Figure 7. In light of the mechanism of plasmon enhanced SHG, the enhancement factor *F*(*ω*,2*ω*) can be defined as [35,36]:

*F*(*ω*,2*ω*) = |*E*(2*ω*)|^2^ |*E*(*ω*)|^4^, (3)

where *E*(*ω*) and *E*(2*ω*) are local electromagnetic field enhancement at fundamental and harmonic frequencies, respectively. From the electromagnetic near-field distribution images of vertical Au nanorod arrays with the diameters of 22 and 41 nm at harmonic and fundamental wavelengths (400 and 800 nm) in Figure 7a,b, it can be found that the maximum fundamental mode enhancement |*E*(*ω*)|^4^ of the vertical Au nanorod array with a diameter of 41 nm is one order of magnitude higher than that of the vertical Au nanorod array with a diameter of 22 nm due to the stronger scattering ability of the larger Au nanorod, while the maximum harmonic mode enhancement |*E*(2*ω*)|^2^ is almost the same for the two Au nanorod arrays. Therefore, the SHG enhancement factor of the vertical Au nanorod array with 41 nm diameter is one order of magnitude higher than that of the Au nanorod array with 22 nm diameter. Although the theoretical simulation result is larger than the experimental one (3.7 times), the experimental result can be verified considering the idealization of the simulation structure, conditions and parameters.

## 4. Conclusions

Vertically aligned Au nanorod arrays with two different diameters of 22 and 41 nm are prepared by evaporation self-assembly method. Their optical properties are dominated by absorption and scattering cross sections, respectively. When coupling with ZnO nanocrystal films, it is found that the vertical Au nanorod array with a larger diameter (41 nm) can enhance the SHG of ZnO nanofilms with an average enhancement factor of about 27.0, while that of the vertical Au nanorod array with a smaller diameter (22 nm) is about 7.3. FDTD simulation shows that the maximum fundamental mode enhancement |*E*(*ω*)|^4^ of the vertical Au nanorod array with a diameter of 41 nm at 800 nm is one order of magnitude higher than that of the vertical Au nanorod array with a diameter of 22 nm due to the stronger scattering ability of the larger Au nanorod, while the maximum harmonic mode enhancement |*E*(2*ω*)|^2^ at 400 nm is almost the same for the two Au nanorod arrays. In the previous work, vertical Au nanorod arrays of various diameters from less than 10 nm to tens of nanometers have been used to enhance luminescence and Raman scattering. Our work shows that the diameter of Au nanorods plays an active role in realizing strong plasmon enhancement of vertically aligned Au nanorod arrays.

## Data Availability

The data used to support the findings of this study are available from the corresponding author upon request.

## Supplementary Materials

The following are available online at https://www.mdpi.com/article/10.3390/nano11102597/s1, Figure S1: SEM images of vertical Au nanorod arrays with organic residues on their surface.
