# Supplementary material for: Plasmon Enhanced Second Harmonic Generation from ZnO Nanofilms on Vertical Au Nanorod Arrays
Source: Nanomaterials (Basel). 2021 Oct 2;11(10):2597. doi: 10.3390/nano11102597 (PMC8539005; doi:10.3390/nano11102597)
Supplement: Supplementary file 1 [file nanomaterials-11-02597-s001.zip › nanomaterials-1374841-supplementary.pdf]

Supplement Materials

Plasmon Enhanced Second Harmonic Generation from ZnO Nanofilms on Vertical Au Nanorod Arrays

Qiang Ma [1], Chengda Pan [1], Yingxian Xue [1], Zhiyun Fang [1], Shiyu Zhang [1], Botao Wu [1,*] and E Wu [1,2]

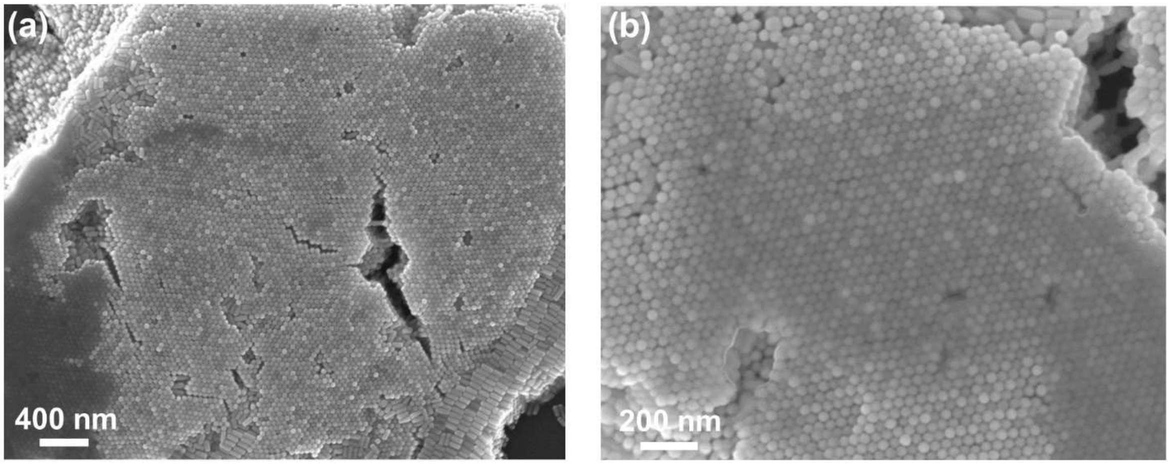

Figure S1. (a) and (b) SEM images of vertical Au nanorod arrays with random organic residues on their surface.
